# Use of balloon angioplasty for patients with intracranial large atherosclerotic acute ischemic stroke and cerebral cavernous malformation: a case report

**DOI:** 10.3389/fneur.2025.1500451

**Published:** 2025-01-22

**Authors:** Yiwan Wang, Tianyu Wang, Jiangmin Liang

**Affiliations:** ^1^Department of Emergency, Taizhou Central Hospital (Taizhou University Hospital), Taizhou, China; ^2^Department of Neurology, Taizhou Central Hospital (Taizhou University Hospital), Taizhou, China; ^3^Department of Gastroenterology, Taizhou Central Hospital (Taizhou University Hospital), Taizhou, China

**Keywords:** balloon angioplasty, intracranial large atherosclerotic, acute stroke, cerebral cavernous malformation, case report

## Abstract

Thus far, clinical data relating to the treatment of cerebral cavernous malformation (CCM) patients with acute stroke (AIS) are incredibly scare due to the low incidence of CCM. Furthermore, the safety profile of using tissue plasminogen activator, the only drug approved for AIS treatment within 4.5 h, remains controversial in patients with CCM. Recently, balloon angioplasty has been reported as a successful treatment for intracranial large atherosclerotic AIS patients. In our department, we treated a patient with intracranial large atherosclerotic AIS and CCM using balloon angioplasty, resulting in a positive outcome. Here, we discuss the safety and efficiency of balloon angioplasty for the treatment of intracranial large atherosclerotic AIS in patients with CCM. In conclusion, we suggest that balloon angioplasty may be a potentially safe and effective treatment for intracranial large atherosclerotic AIS patients with CCM. However, further research is needed to explore the use of mechanical revascularization in AIS patients with CCM.

## Introduction

Cerebral cavernous malformation (CCM) is a major cause of intracranial hemorrhage (ICH), with an incidence of only 0.5% (1). A previous retrospective analysis of over 20,000 autopsies found that 95% of CCM cases were asymptomatic (1). Due to its low incidence, there is a considerable lack of clinical data relating to the treatment of patients with acute ischemic stroke (AIS) and CCM.

AIS is a major cause of disability and death, and its treatment has evolved considerably over recent decades. At present, intravenous tissue plasminogen activator is the only drug proven to be beneficial for AIS patients within 4.5 h of symptom onset. However, several articles have reported cases of symptomatic intracranial hemorrhage (sICH) and even death in CCM patients treated with intravenous tissue plasminogen activator (2).

Endovascular therapy, such as stenting and balloon angioplasty, are becoming increasingly important for the treatment of stroke, especially for patients with contraindications for intravenous thrombolytic therapy. However, until now, the safety and efficiency of balloon angioplasty for acute stroke patients with CCM has not been specifically investigated. Herein, we present the case of an AIS patient with intracranial large artery atherosclerosis and CCM who received balloon angioplasty.

### Case report

A 58-year-old male, was admitted to our neurological department on the 4th of June 2022 due to the fact that 2.5 h previously, he suffered a sudden onset of slurred speech which lasted for approximately 5 min, but then recovered spontaneously. However, these symptoms recurred approximately 10 min later; on this occasion, the patient did not recover. His medical history included hypertension, managed with 2.5 mg/day of amlodipine, gastrointestinal hemorrhage within the last 3 weeks, an ischemic stroke within 3 months previously without sequelae (26th of April 2022), and radical rectal cancer surgery 6 months previously.

**Figure 1:**
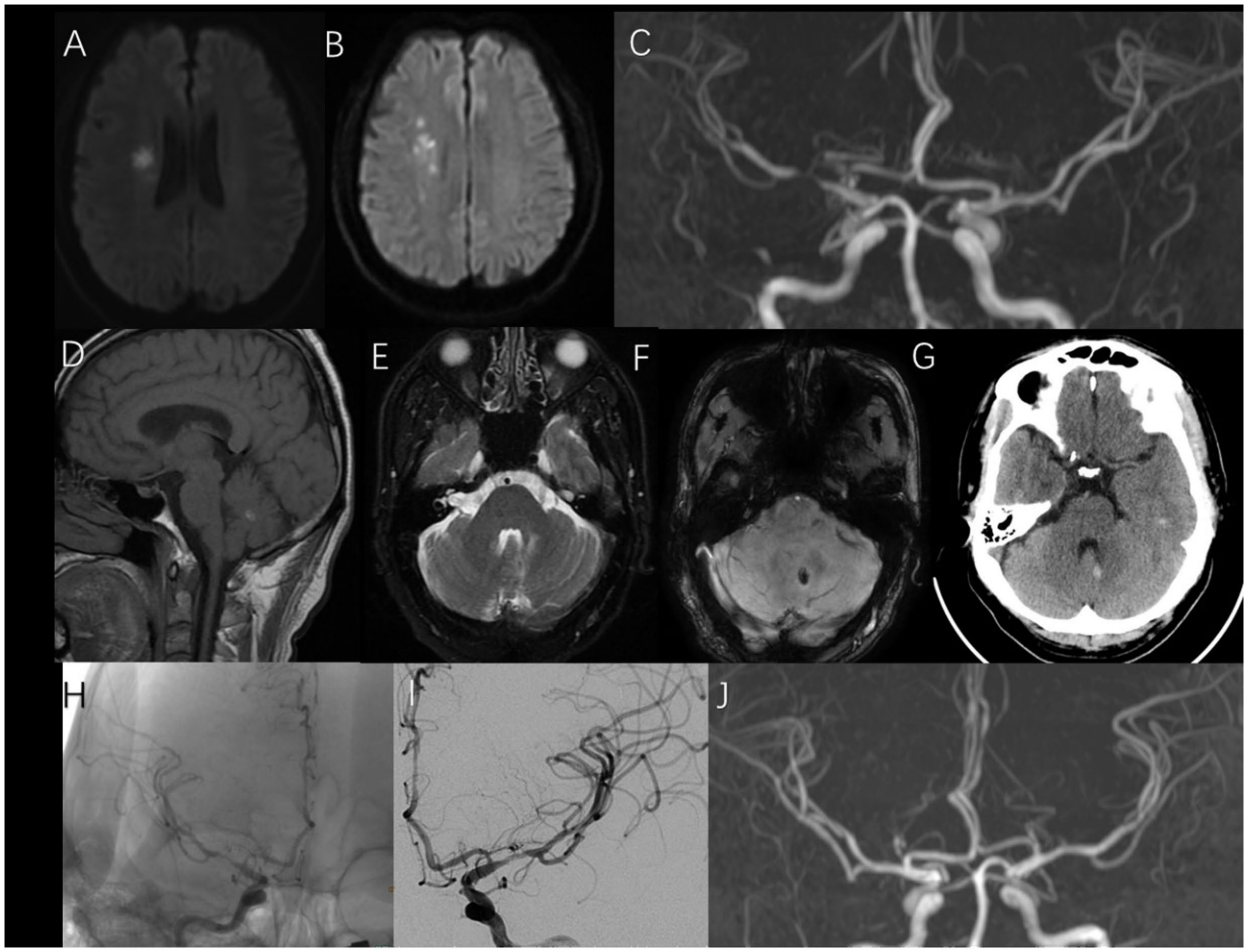
**(A, B)** Ischemic stroke lesion in the paraventricular region; **(C)** Stenosis in the right middle cerebral artery; **(D–G)**: Cerebral cavernous malformation in the left cerebellum **(D)**: T1, **(E)**: T2, **(F)**: susceptibility-weighted imaging, **(G)**: computed tomography; **(H)**: Stenosis in the right middle cerebral artery; **(I)**: Successful dilation in the right middle cerebral artery; **(J)**: Right middle cerebral artery in follow-up period.

Upon arrival, neurological examination revealed mild left facial paralysis, dysarthria, with a score of 3 on the National Institutes of Health Stroke Scale (NIHSS). Magnetic resonance imaging (MRI) and magnetic resonance angiography identified an ischemic stroke lesion in the paraventricular region, along with stenosis in the right middle cerebral artery, left vertebral artery, and right posterior cerebral artery (Figures 1A–C). A lesion in the left cerebellum was revealed and evaluated by MRI, susceptibility-weighted imaging and computed tomography, performed at our hospital on August 5th, 2020, confirming the presence of a CCM (Figures 1D–G). Arterial spin labeling further indicated hypoperfusion in the right cerebral hemisphere. Due to contraindications for intravenous thrombolytic therapy, we considered mechanical revascularization. Surgery was commenced approximately 180 min after the onset of symptoms. During the procedure, we used a 5 French guiding catheter and a synchro2 micro-catheter. Stenosis in the right middle cerebral artery was confirmed (Figure 1H). Thus, we performed balloon pre-dilation with 2.0 × 15-mm and 2.5 × 15-mm balloons in the M1 segment and administered with tirofiban. Ten minutes later, we achieved successful dilation with a grade 2b thrombolysis on the cerebral infarction scale (Figure 1I); therefore, stenting and mechanical thrombectomy were not required. During hospitalization, the patient showed as “++” for fecal occult blood, thus suggesting that he had suffered from a gastrointestinal hemorrhage. His hemoglobin level was within the normal range, at 153 g/L. Postoperatively, we initiated aspirin. During hospitalization, his symptoms improved slightly, reflected by an NIHSS score of 2. The patient was subsequently discharged 6 days later.

During the follow-up period, we re-performed magnetic resonance angiography (4 months after discharge); this scan did not reveal any evidence of restenosis (Figure 1I). The patient was subsequently followed-up on the 16th of October 2024; no further complaints were reported.

## Discussion

To the best of our knowledge, this is the first article to discuss the safety and efficiency of balloon angioplasty for the treatment of intracranial large atherosclerosis AIS patients with CCM. This is also the first study to report mechanical revascularization in such patients.

CCM is an important risk factor for ICH, despite its low prevalence of approximately 0.5% (1). CCM is a cerebrovascular malformation that is usually located in the brain, spinal cord, or rarely, the dura. Generally, CCM can be divided into two types: familial or sporadic (3). Most patients with CCM are asymptomatic, while others experience certain symptoms such as headaches, seizures, or focal neurological deficits, often associated with ICH (3). A retrospective study, performed over 8 years and involving 133 patients, reported an overall hemorrhage rate of 2.19% annually (4). Thus, the risk factor for hemorrhage in CCM has already been addressed.

Interestingly, a previous cohort study found that the administration of anti-thrombotic agents (such as anticoagulants or antiplatelet agents) did not increase the frequency of CCM-associated ICH (5). Furthermore, a previous meta-analysis suggested that anti-thrombotic therapy could reduce the incidence of ICH in CCM patients (6). Anti-thrombotic treatment was also used to treat our present patient with no evidence of hemorrhage during follow-up. This has led some researchers to hypothesize that ICH may result from thrombus formation in associated venous malformations or within the dilated caverns of CCMs due to slow blood flow (6).

However, due to limited clinical data, the safety of tissue plasminogen activator in the treatment of AIS patients with CCM remains uncertain. A recent study, including 30 patients, reported that the risk of sICH in AIS patients with CCM after thrombolysis was relatively higher than regular patients (10% *versus* 3.5%) (2). Although this finding may be associated with publication and reporting bias, along with a small sample size, this finding highlights the need for caution with regards to the risk of hemorrhage. Furthermore, indications for thrombolysis are more strict than mechanical revascularization, including a shorter treatment window.

The treatment of stroke has evolved considerably since coronary angioplasty was introduced into clinical practice by Gruntzig et al. (7). Numerous studies have proven the safety and efficiency of balloon angioplasty for intracranial atherosclerotic stenosis (8–11). The combination of a balloon guide catheter and stent retrievers has already been recommended by guidelines produced by the American Heart Association/American Stroke Association (COR IIa; LOE C-LD) (12). However, the application of direct balloon angioplasty to treat stroke is not specifically mentioned in these guidelines. A recent retrospective cohort study involving 68 patients suggested that direct balloon angioplasty might represent an effective and safe method to treat AIS patients with intracranial large atherosclerosis (8). However, balloon angioplasty is associated with certain drawbacks, including restenosis (8). In another study, Chen et al. reviewed the ratio of restenosis from four cohorts and found that this key ratio ranged from 0 to11.8% (Table 1) (8). However, our present patient did not experience restenosis during the follow-up period. Furthermore, in these previous articles, intravenous tissue plasminogen activator was not used as a criterion for exclusion, and the occurrence of sICH ranged from 0–4.4% (Table 1), thus suggesting that balloon angioplasty may not increase the risk of hemorrhage (8). However, patients with intracranial atherosclerosis were the main research targets described in these previous articles; in contrast, patients with other Acute Stroke Treatment classifications, such as cardioembolism, were not fully discussed. Furthermore, our present case was disadvantaged because the patient had mild symptoms; thus, our case cannot provide strong support with regards to efficiency. Another disadvantage is the retrospective design of our case report.

**Table 1:** The rates of sICH and restenosis in four different published cohorts.

Authors	Cohort patients	IV-tPA	sICH	Restenosis
Zhang et al.	5	2	0	0
Kim et al.	30	4	0	3 (10%)
Kim et al.	68	30	3 (4.4%)	8 (11.8%)
Chen et al.	41	41	1 (2.4%)	–

IV-tPA, intravenous tissue plasminogen activator; sICH, symptomatic intracranial hemorrhage.

Finally, it is important to consider that clinical data relating to the treatment of AIS patients with CCM is very scare. Our present case suggested that balloon angioplasty might be a potential therapy for intracranial large atherosclerosis AIS patient with CCM. In clinical practice, each AIS patient with CCM should undergo an individual, comprehensive evaluation by a clinical practitioner. Given the potentially higher risk of hemorrhage, balloon angioplasty may be superior for the treatment of thrombolysis in intracranial large atherosclerosis AIS patients with CCM. Furthermore, since balloon angioplasty has been primarily tested in patients with intracranial atherosclerosis, other mechanical revascularization methods should be considered for CCM patients with non-intracranial atherosclerosis AIS. Future research should evaluate the risk–benefit stratification of the use of mechanical revascularization for AIS patients with CCM.

## Conclusion

This is the first study to report the use of balloon angioplasty in treating intracranial large atherosclerotic AIS patients with CCM. Our findings suggested that balloon angioplasty might be a safe and effective treatment for these patients, potentially offering advantages over thrombolysis. Future research should investigate the use of mechanical revascularization for AIS patients with CCM.

## Data Availability

The datasets presented in this article are not readily available because of ethical and privacy restrictions. Requests to access the datasets should be directed to the corresponding authors.
